# Human Pluripotent Stem Cell-Derived Neural Crest Cells for Tissue Regeneration and Disease Modeling

**DOI:** 10.3389/fnmol.2019.00039

**Published:** 2019-02-22

**Authors:** Akshaya Srinivasan, Yi-Chin Toh

**Affiliations:** ^1^Department of Biomedical Engineering, National University of Singapore, Singapore, Singapore; ^2^Singapore Institute for Neurotechnology (SINAPSE), National University of Singapore, Singapore, Singapore; ^3^NUS Tissue Engineering Program, National University of Singapore, Singapore, Singapore; ^4^Biomedical Institute for Global Health, Research and Technology, Singapore, Singapore

**Keywords:** neural crest, disease model, tissue regeneration, pluripotent stem cell, neurocristopathy

## Abstract

Neural crest cells (NCCs) are a multipotent and migratory cell population in the developing embryo that contribute to the formation of a wide range of tissues. Defects in the development, differentiation and migration of NCCs give rise to a class of syndromes and diseases that are known as neurocristopathies. NCC development has historically been studied in a variety of animal models, including xenopus, chick and mouse. In the recent years, there have been efforts to study NCC development and disease in human specific models, with protocols being established to derive NCCs from human pluripotent stem cells (hPSCs), and to further differentiate these NCCs to neural, mesenchymal and other lineages. These *in vitro* differentiation platforms are a valuable tool to gain a better understanding of the molecular mechanisms involved in human neural crest development. The use of induced pluripotent stem cells (iPSCs) derived from patients afflicted with neurocristopathies has also enabled the study of defective human NCC development using these *in vitro* platforms. Here, we review the various *in vitro* strategies that have been used to derive NCCs from hPSCs and to specify NCCs into cranial, trunk, and vagal subpopulations and their derivatives. We will also discuss the potential applications of these human specific NCC platforms, including the use of iPSCs for disease modeling and the potential of NCCs for future regenerative applications.

## Introduction

**FIGURE 1:**
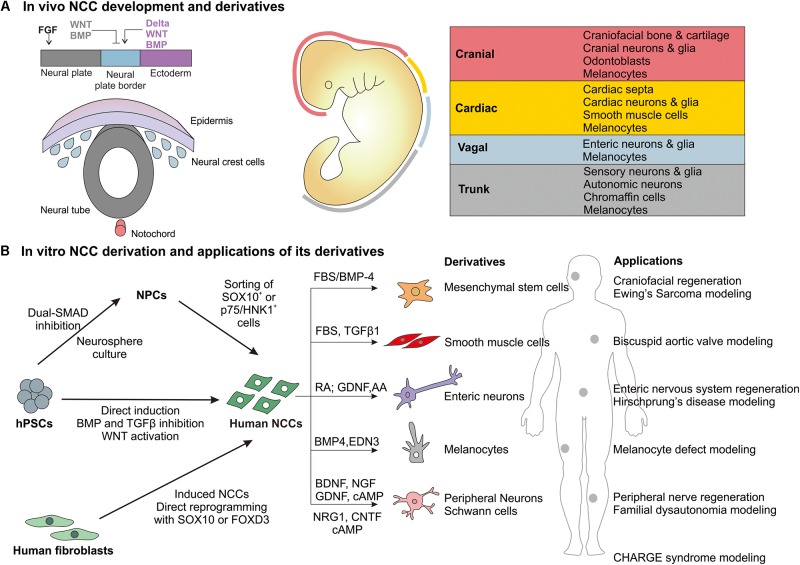
Overview of *in vivo* NCC development and derivatives and *in vitro* derivation of human NCCs and major applications. **(A)** During gastrulation, the neural plate border is specified by BMP, WNT, FGF, and Notch/Delta signaling from the surrounding neural plate, non-neural ectoderm and mesoderm. NCCs are specified at the neural plate border region and then reside in the dorsal portion of the neural tube. Following neural tube closure, they undergo an epithelial-mesenchymal transition and migrate along the anterior-posterior axis of the embryo to give rise to different derivatives based on the region (cranial, cardiac, vagal, or trunk) (Milet and Monsoro-Burq, 2012b; Simoes-Costa and Bronner, 2015; Gandhi and Bronner, 2018). **(B)** The major approaches by which human NCCs are derived *in vitro* from hPSCs and differentiated to selected derivatives. The potential applications of these derivatives in regenerative medicine and disease modeling.

Neural crest cells (NCCs) are transient, migratory stem cells that originate from the neural tube and migrate to different embryonic tissues to give rise to a wide variety of cell types (Le Douarin et al., 2004). They form ectodermal derivatives, such as sensory and enteric neurons, Schwann cells, as well as mesenchymal derivatives (Le Douarin and Dupin, 2003). Thus, NCCs have been widely studied in animal models to elucidate their role in a range of neurocristopathies involving the craniofacial skeleton and the peripheral nervous system. The advent of techniques for the derivation of human neural crest cells (hNCCs) from human pluripotent stem cells (hPSCs), has not only enabled the understanding of NCC development and disease in a human-specific context but also opened new opportunities for therapeutic applications. This review will highlight the state-of-the-art protocols used to derive hNCCs from hPSCs and discuss opportunities and challenges in the applications of hNCCs in disease modeling and tissue regeneration (Figure 1).

## Derivation of Human NCCs and Its Derivatives From hPSCs

During embryonic development, NCCs are specified at the neural plate border (NPB) region before they undergo an epithelial-mesenchymal transition and migrate out of the neural tube (Figure 1A). It has been shown that NCC induction at the NPB relies on BMP, WNT, Notch/Delta, and FGF signaling emanating from the surrounding embryonic tissues (Rogers et al., 2012). Based on these molecular developmental programs, researchers have developed increasingly specific and efficient protocols to derive an NCC population from hPSCs *in vitro* as highlighted below.

### Directed NCC Induction Using Small Molecules

Since NCCs are specified adjacent to neural plate cells *in vivo*, early strategies sought to derive a mixed neural precursor cell (NPC) population and enrich the NCC subpopulation. NPC induction methods include stromal cell co-culture (Pomp et al., 2005; Lee et al., 2007; Jiang et al., 2009), neurosphere culture (Brokhman et al., 2008; Bajpai et al., 2010) or defined monolayer induction of neural rosettes using dual-SMAD inhibition of BMP and Activin A/Nodal signaling (Chambers et al., 2009). Limited p75^+^/HNK-1^+^ NCCs could be purified from the periphery of these neural rosettes (Lee et al., 2010). These NPC-based protocols were highly inefficient and variable because NCCs represented a small subset of NPCs, and groups used different combinations of NCC markers such as p75, HNK-1, and SOX10 to isolate NCCs (Milet and Monsoro-Burq, 2012a). Also, the role of the stromal cells/cell aggregates/neural rosettes in inducing NCC formation was unclear, limiting their utility in the study of the molecular mechanisms of NCC development.

To overcome these limitations, researchers sought to achieve directed and specific NCC induction in adherent culture using a defined system. NCC development *in vivo* requires the activation of canonical WNT signaling and well as intermediate levels of BMP signaling, after an initial inhibition to establish neural plate identity (Pla and Monsoro-Burq, 2018). Mimicking this, researchers used combinations of small molecule activators of WNT signaling and inhibitors of BMP and Activin/Nodal signaling to achieve directed NCC differentiation from both hESCs and iPSCs (Menendez et al., 2011, 2013; Mica et al., 2013). While Mica et al. found a short pulse of BMP inhibition to be essential for NCC specification, Menendez et al. found it unnecessary in their hPSC culture system. This controversy on the requirement of BMP inhibition for NCC specification was addressed by Hackland et al., who demonstrated that the levels of endogenous BMP signaling differ across various hPSC cell lines and *in vitro* culture systems. They could achieve the precise intermediate BMP level required for robust NCC derivation by a top-down inhibition of BMP signaling in a completely defined culture system (Hackland et al., 2017). The directed induction approach is the most commonly used method to derive NCCs from hPSCs, and can generally achieve a high derivation efficiency, ranging from 40 to 90%. However, different groups employ different markers (e.g., SOX10^+^ or p75^+^/HNK1^+^) to identify NCCs and may not be isolating identical NCC populations.

### Transcription Factor-Based Reprogramming

Neural crest cells have also been directly induced from fibroblasts by reprogramming with a single transcription factor SOX10, in the presence of environmental cues including a WNT activator (Kim et al., 2014). This system could be used to derive induced NCCs (iNCCs) directly from human patient fibroblasts. Direct reprogramming from fibroblasts allows for the generation of patient-specific NCCs as well as potentially reducing the length of *in vitro* culture before the iNCCs can be administered clinically. Another method to induce NCCs from fibroblasts involves the use of a chitosan substrate to effect the gene transfer of FOXD3, leading to increased NCC marker expression and the ability to rescue impaired neural function in a zebrafish model (Tseng et al., 2016). While this system provides a non-viral alternative for reprogramming to iNCCs, the exact nature of the induced cells was not fully defined due to incomplete characterization and lack of *in vitro* differentiation studies. Further work is required to reprogram iNCCs using non-viral methods to enable their use in clinical applications.

### Regional Specification of Derived NCCs

Neural crest cells arise from four distinct regions of the anterior-posterior axis of the neural tube: cranial, cardiac, trunk, and vagal (Achilleos and Trainor, 2012). These NCC sub-populations express specific markers and have distinct differentiation potentials (Figure 1A). The dual-SMAD inhibition/WNT activation protocols tend to derive a HOX-negative NCC population that is disposed toward anterior (cranial) over posterior (vagal) identity (Mica et al., 2013). While early inhibition of BMP signaling is necessary to establish NCC identity, late supplementation of BMP-4 during NCC derivation can enhance cranial identity, as indicated by the upregulation of cranial-specific *DLX* genes (Mimura et al., 2016). These cranial NCCs also expressed pharyngeal mesenchymal genes and showed osteogenic and chondrogenic differentiation potential *in vitro*. This can be advantageous as a defined culture system to generate mesenchymal derivatives from hNCCs as compared to the use of undefined serum to drive NCCs into mesenchymal lineages.

Retinoic acid (RA) and FGF-2 are known to be caudalizing factors during neural development (Stuhlmiller and García-Castro, 2012), and thus were utilized to posteriorize hPSC-derived NCCs as well. Vagal NCCs could be specified by the addition of RA or FGF-2 to a NC induction protocol (Mica et al., 2013). Multiple studies then showed that RA could posteriorize hPSC-derived NCCs to enteric NCCs (ENCCs), which expressed vagal-specific *HOXB* genes and could differentiate into various enteric neuron subtypes (Fattahi et al., 2016; Schlieve et al., 2017). Sequential treatment with RA and BMP could generate PHOX2B^+^ trunk NCCs, which could differentiate into sympathoadrenal cells (Huang et al., 2016). This mimicked the *in vivo* induction of sympathoadrenal trunk NCCs by BMP signaling from the dorsal aorta (Saito et al., 2012). The emergence of these protocols to define regional NCC identity will be useful in determining intermediate cell populations during differentiation to NCC derivatives. Currently, it appears that many groups use either FBS/BMP-4 or RA to direct anterior or posterior NCC specification before differentiating NCCs to end-point derivatives (Figure 1B) using protocols optimized for other cell types. Widespread adoption and further development of defined protocols to drive and characterize region-specific NCCs will be beneficial to create more defined culture systems. This will enable the future use of NCC derivatives for tissue regeneration applications.

## Applications of hPSC-Derived NCCs for Disease Modeling

Human-specific NCCs provide an invaluable resource to complement animal models in the understanding of neurocristopathies. Therefore, a major application of hPSC-derived NCCs to date is in the modeling of various neurocristopathies (Table 1). The following section highlights notable neurocristopathies whereby the generation of either patient-specific iPSC-derived NCCs or genetically modified hESCs bearing specific genetic mutations have been used to mimic clinically relevant NCC dysfunctions and discover novel molecular mediators.

**Table 1:** Applications of hPSC-derived NCCs in disease modeling and tissue regeneration.

NCC derivatives	Intended applications	Cell source	NCC derivation method	Key outcomes	Reference
None	Disease modeling-CHARGE syndrome	CHD7 knockdown H9 hESC cell line	Neurosphere culture followed by isolation of migratory NCCs	Reduced formation of multipotent, migratory *TWIST1*^+^ NCCs	Bajpai et al., 2010
		iPSCs from CHARGE patient-derived fibroblasts	Methods of Lee et al. (2009) and Bajpai et al. (2010)	Defective delamination *in vitro* and migration *in vitro* and *in vivo* by CHARGE NCCs	Okuno et al., 2017
Mesenchymal stem cells (MSCs)	Disease modeling-Ewing’s Sarcoma	H9 hESC cell line	Ectopic expression of EWS-FLI1 in p75^+^ NCCs isolated after PA6 co-culture	Transition of EWS-FLI1 MSCs to a more primitive state, p16 repression	von Levetzow et al., 2011
	Tendon regeneration	iPSCs from human BMSCs	p75^+^ migratory NCCs isolated after cell aggregate culture	Enhanced healing by NCCs delivered in fibrin gel compared to control in rat patellar tendon window defect model	Xu et al., 2013
	Cartilage regeneration	414C2 human iPSC cell line	WNT activation and TGF-β inhibition, followed by 10% FBS (MSC induction)	Poor defect repair by NCC cell sheet compared to control BMSC cell sheet in rat femoral osteochondral defect model	Chijimatsu et al., 2017
Smooth Muscle Cells (SMCs)	Disease modeling- Bicuspid aortic valve (BAV)	iPSCs from BAV patient-derived PBMCs	p75^+^/HNK-1^+^ NCCs isolated after dual-SMAD inhibition	BAV SMCs had impaired contractility and increased mTOR signaling	Jiao et al., 2016
Enteric neurons	Disease modeling- Hirschsprung’s disease (HSCR)	iPSCs from HSCR patient-derived fibroblasts and RET mutant IMR90 iPSCs	Dual-SMAD inhibition, WNT activation, RA treatment to derive p75^+^/CD49^+^ ENCCs	Defective migration and neuronal differentiation in HSCR NCCs; identification of mutations associated with HSCR and correction with CRISPR/Cas9	Lai et al., 2017
	Enteric Nervous System (ENS) Regeneration Enteric Nervous System (ENS) Regeneration	H9 hESCs and iPSC cell lines	Dual-SMAD inhibition, WNT activation, RA treatment to derive p75^+^/CD49^+^ ENCCs	Extensive migration of grafted ENCCs delivered in 70% matrigel; rescue of disease-related mortality in Hirschsprung disease mice (*Ednrb*^s-l/s-l^)	Fattahi et al., 2016
		H9 hESCs and WTC iPSC cell lines	Dual-SMAD inhibition, WNT activation, RA treatment to derive p75^+^/CD49^+^ ENCCs	Establishment of ganglia, neuronal repopulation, neuron-dependent contractility by 3-D spheroids of ENCCs implanted in human tissue-engineered small intestine	Schlieve et al., 2017
		iPSCs from human dermal and embryonic fibroblasts	p75^+^/HNK-1^+^ migratory NCCs isolated after aggregate culture; co-culture with gut explants to induce enteric neurons	Longitudinal migration in E5 chick hindgut, migration toward myenteric and submucosal regions in SCID mice upon engraftment of NCC spheres	Li et al., 2018
		H1 and H9 hESCs, WTC iPSC cell lines	Neurosphere culture followed by isolation of migratory NCCs	Migration of ENCCs into mesenchyme; neuronal and glial differentiation upon mechanical aggregation of ENCCs with tissue-engineered human intestinal organoids	Workman et al., 2017
Peripheral Neurons and Schwann Cells	Disease modeling- Familial dysautonomia (FD)	iPSCs from FD patient-derived fibroblasts	p75^+^/HNK-1^+^ NCCs isolated after MS5 co-culture	*IKBKAP* splicing defect, reduced migration and neuronal differentiation of FD NCCs	Lee et al., 2009
		Normal and FD patient fibroblasts	Direct NCC reprogramming by SOX10 overexpression and WNT activation	*IKBKAP* splicing defect, reduced iNCC induction and migration in FD iNCCs	Kim et al., 2014
	Peripheral Nerve Regeneration	Human iPSC cell lines	LNGFR1 (p75) ^+^/THY1^+^ migratory NCCs isolated after neurosphere culture	Promotion of axonal regrowth and remyelination in silicone nerve conduit in NCC group in mouse sciatic nerve defect model	Kimura et al., 2018
		hESCs	p75^+^ NCCs isolated after dual-SMAD inhibition and WNT activation	Robust regeneration throughout the trimethylene carbonate 𝜀-caprolactone nerve conduit in NCC group in rat sciatic nerve injury model	Jones et al., 2018
		H9 hESCs	Dual-SMAD inhibition and WNT activation	Therapeutic efficacy of NCC filled poly (𝜀-caprolactone) and ethyl ethylene phosphate nerve conduits reduced with increased passage number in rat sciatic nerve injury model	Du et al., 2018
Melanocytes	Disease modeling- Hermansky-Pudlak (HP) and Chediak-Higashi (CH) syndromes	iPSCs from HP and CH patient-derived fibroblasts	Dual-SMAD inhibition and WNT activation followed by isolation of SOX10^+^/cKit^+^ melanocyte precursors	Loss of pigmentation and reduction in mature melanosomes in different degrees in CH and HP NCC derived melanocyte clones	Mica et al., 2013


*hESCs: human embryonic stem cells; PBMCs: peripheral blood mononuclear cells.*

### CHARGE Syndrome

CHARGE syndrome is an acronym for a complex combination of congenital abnormalities including malformations of the craniofacial skeleton, peripheral nervous system, eyes, ears, and heart. It is often associated with mutations in the *CHD7* gene, which is postulated to cause NCC dysfunction (Sanlaville and Verloes, 2007). *CHD7* knockdown in hESCs affects the formation of multipotent, migratory NCCs by diminishing the expression of NC specifiers *TWIST1*, *SOX9*, and *SLUG* (Bajpai et al., 2010). Another study derived NCCs from CHARGE patient-derived iPSCs, which also showed defective delamination, migration and motility *in vitro* and defective migration *in vivo* when implanted into a chick embryo (Okuno et al., 2017). Therefore, *CHD7* mutations in CHARGE syndrome result in defects in NCC migration, although the underpinning molecular mechanism remains to be elucidated.

### Ewing’s Sarcoma

Ewing’s sarcoma family tumors (ESFT) are common malignant bone and soft tissue tumors, whose genetic hallmark involves the expression of an EWS-FLI1 fusion gene due to chromosome translocation (Paulussen et al., 2001). While their cellular origin remains elusive, one popular hypothesis implicates NCCs as the source of these cells (Tu et al., 2017). Von Levetzow et al. demonstrated that hESC-derived NCCs and NCC-derived MSCs were permissive for EWS-FLI using lentiviral transduction. Expression of EWS-FLI1 pushed NCC-derived MSCs to a more primitive NCC state and led to the loss of cellular senescence and repression of p16 (von Levetzow et al., 2011). They found that ESFT are genetically closely related to NCCs, supporting the hypothesis that some malignant ESFT cells may develop from NCC-derived cells. The use of this hESC-derived NCC model also helped to delineate the mechanism of oncogene tolerance in these cells.

### Hirschsprung’s Disease

Hirschsprung’s disease (HSCR) is caused by the defective migration of ENCCs in the gut, leading to loss of peristaltic activity, causing bowel obstruction and megacolon. The severity of the phenotype is determined by the length of the aganglionic segment- short (S-HSCR), long (L-HSCR) or total colonic aganglionosis (Amiel et al., 2008). While HSCR is genetically heterogeneous, mutations in the receptor tyrosine kinase RET are implicated in many cases. Lai et al. generated iPSCs from HSCR patients as well as CRISPR-Cas9 edited RET mutant iPSC lines, and demonstrated that both HSCR and RET-mutant ENCCs showed defective neuronal differentiation and migration compared to control ENCCs (Lai et al., 2017). They identified a novel mutation in the vinculin gene associated with S-HSCR, and corrected this mutation using CRISPR/Cas9 to restore ENCC function. This study demonstrates the great potential of hPSC-based *in vitro* assays to identify novel disease-associated mutations with high power. This will be useful in the study of HSCR as its genetic etiology is still not completely known.

### Familial Dysautonomia

Familial dysautonomia (FD) is a rare but fatal, hereditary sensory and autonomic neuropathy usually caused due to a point mutation in the *IKBKAP* gene (Slaugenhaupt et al., 2001). FD is known to affect NCCs and cause degeneration of peripheral neurons. Lee et al. first reprogrammed iPSCs from FD patient fibroblasts and differentiated them to NCCs, while Kim et al. directly reprogrammed FD patient fibroblasts to iNCCs by SOX10 overexpression (Lee et al., 2009; Kim et al., 2014). In both studies, FD NCCs showed lower levels of normal *IKBKAP* transcripts, reduced migration and lower neuronal differentiation efficiency compared to control NCCs. Lee et al. went on to identify kinetin as a candidate drug to rescue aberrant *IKBKAP* splicing, while Kim et al. shed light on a previously unknown aspect of FD pathogenesis- aberrant splicing in other genes such as *PAX3* and *MEF2C* in FD iNCCs.

### Hermansky-Pudlak Syndrome and Chediak-Higashi Syndrome

Pigment producing melanocytes in the skin arise from NCCs during development. Mica et al. developed a protocol involving timed exposure to WNTs, BMPs, and EDN3s for the sequential specification of NCCs, melanoblasts and mature melanocytes (Mica et al., 2013). They then derived iPSCs from patients with Hermansky-Pudlak syndrome (HP) and Chediak-Higashi syndrome (CH), both of which cause defects in melanocyte vesicle formation and trafficking. Melanocytes derived from HP and CH NCCs showed different degrees of pigmentation loss and reduction in melanosome number and size, corresponding to the expected disease phenotype.

## Applications of hPSC-Derived NCCs in Regenerative Medicine

Due to their wide differentiation potential, unlimited numbers, and developmental relevance to many tissues, hPSC-derived NCCs are a promising stem cell source for tissue regeneration and as therapies for neurocristopathies. iPSC-derived NCCs are a potentially autologous cell source that can overcome immune-compatibility issues. The preliminary investigations that have been done to assess the regenerative potential of hPSC-derived NCCs are discussed below (Table 1).

### Bone, Cartilage, and Tendon

Currently, mesenchymal stem cells (MSCs) derived from bone marrow and adipose tissues are the paradigm cell source for the regeneration of craniofacial bone and cartilage (Yamano et al., 2012; Tollemar et al., 2016). However, since a significant portion of craniofacial mesenchymal tissues originate from NCCs, hPSC-derived NCCs are a promising alternative cell source for craniofacial bone and cartilage tissue engineering. hPSC-derived NCCs can be induced into MSCs either by fetal bovine serum (FBS) or by BMP-4 treatment (Mimura et al., 2016). Although FBS treatment is more prevalent, the use of undefined serum in MSC induction medium will limit future clinical applications. Mechanical cues such as substrate stiffness have recently been shown to modulate the differentiation potential of NCC-derived MSCs (Srinivasan et al., 2018). To date, only a handful of studies have evaluated the regenerative potential of NCC-derived MSCs. Chijimatsu et al. showed that although MSC-like cells differentiated from iPSC-derived NCCs showed chondrogenic ability *in vitro*, they had very limited repair efficiency in a rat osteochondral defect model (Chijimatsu et al., 2017). On the other hand, Xu et al. demonstrated that iPSC-derived NCCs exhibited enhanced tendon healing compared to the acellular control group in a rat patellar tendon defect model (Xu et al., 2013). Such differences in the regenerative efficiencies of NCC-derived MSCs are likely due to variations in the differentiation protocols for NCCs and MSCs as well as the choice of defect models. In both studies, the use of a single marker p75 to isolate NCCs is problematic, as studies have shown that p75 is not exclusive to NCCs and is widely expressed in the embryonic tissues (Betters et al., 2010). Further work is required to develop chemically defined protocols to drive human NCCs into different mesenchymal lineages, and comprehensively benchmark their regenerative potential to mesodermal MSC sources.

### Enteric Nervous System

As the enteric nervous system (ENS) is derived from the neural crest (Iwashita et al., 2003), NCC-derived enteric neurons are an obvious choice of cell source for ENS regeneration. ENCC precursors derived from hPSCs were able to colonize postnatal and adult mouse colons upon *in vivo* engraftment and showed extensive migration (Fattahi et al., 2016). The ENCCs were also able to rescue disease-related mortality in a genetic mouse model of HSCR (*Edrnb^s-l/s-l^* mice). Two recent studies demonstrated the use of hPSC-derived ENCCs to populate human intestinal organoids with an ENS (Schlieve et al., 2017; Workman et al., 2017). Schlieve et al. demonstrated that the implantation of ENCCs into their tissue-engineered small intestine derived from human intestinal organoids (HIO-TESI) led to the repopulation of an ENS in the HIO-TESI system and the establishment of neuron-dependent motility. Another study showed that upon transplantation of NCC spheres into E5 chick embryonic hindgut, they showed ganglial organization within submucosal and myenteric regions and longitudinal migration (Li et al., 2018). Taken together, these studies suggest that hPSC-derived ENCCs are a promising cell source for treating human ENS disorders.

### Peripheral Nerves

Peripheral nerve regeneration using primary Schwann cells is very difficult due to limited cell numbers, long culturing times and invasive harvesting techniques (Walsh and Midha, 2009). Thus, stem cell sources such as NCCs that can differentiate into Schwann cells have emerged as a promising alternative. Multiple studies have demonstrated the ability of NCCs to differentiate into Schwann cells and repair peripheral nerve defects when implanted as a nerve graft including a scaffold. Using rat or mice sciatic nerve injury models, these studies showed that grafted NCCs survived and promoted axonal regeneration in the artificial nerve conduits (Du et al., 2018; Jones et al., 2018; Kimura et al., 2018). While Jones et al. derived NCCs from hESCs that were only p75^+^, Kimura et al. derived a LNGFR^+^ (p75), THY1^+^ (CD90, a common MSC marker) NCC population from iPSCs, likely selecting for an MSC sub-population. Thus, the different markers used in these studies probably led to the isolation of two different cell populations, which may affect regeneration via different mechanisms. The *in vitro* culture duration of hPSC-derived NCCs, as indicated by the passage number, is also found to impact of their therapeutic efficacy as indicated by Schwann cell differentiation, survival and axonal growth (Du et al., 2018). This finite expansion window presents a practical constraint in the application of hPSC-derived NCCs in peripheral neuronal regeneration applications.

## Conclusion and Future Perspectives

This extensive body of work to induce the formation of NCCs and its derivatives *in vitro* has enabled the use of hPSC-derived NCCs for applications including disease modeling and tissue regeneration. Studies involving patient-derived and genetically modified NCCs have already broadened our understanding of NCC development and disease. The use of iNCCs reprogrammed directly from patient fibroblasts will likely advance this process further. So far, most of these studies have used 2-D monolayer culture systems. As signaling from surrounding tissues is so critical in NCC development *in vivo*, the development of 3-D organotypic models containing multiple cell types would better replicate the *in vivo* environment. This will help us gain a better understanding of NCC disease development in human-specific models. Moving forward, the use of human NCC-based models to test developmental toxicity and screen for possible human teratogens is a likely prospect. This can be enabled by the development of scalable, cost-effective and biomimetic models of NCC development.

The preliminary studies on the use of hPSC-derived NCCs for tissue regeneration show the great promise of these cells due to their wide differentiation potential and large cell numbers. However, more defined differentiation regimes optimized for specifying NCCs into specific lineages are needed to reliably produce purified cell populations. These should be characterized not only by marker expression, but also transplantation to test functional capability. The chief issue that impedes the clinical translation of NCC-derived cells for regeneration is the safety concern with the use of hPSC-derived cells. While a few studies have shown that the implantation of hPSC-derived NCCs in animal models did not cause teratoma formation (Wang et al., 2011; Chijimatsu et al., 2017), thorough strategies to prevent uncontrolled proliferation are required to prevent any risk. Also, there are still unanswered questions regarding the relevance of a cell source’s developmental origin in regenerative medicine. It remains to be seen whether, for example, the use of developmentally relevant NCC-derived MSCs for craniofacial regeneration improves the therapeutic efficacy over other MSC sources, such as mesodermal derived bone marrow MSCs.

## Author Contributions

AS and Y-CT conceived and wrote this manuscript.

## Conflict of Interest Statement

The authors declare that the research was conducted in the absence of any commercial or financial relationships that could be construed as a potential conflict of interest.
